# Religiosity and Attitudes towards Health, Disease, Death and the Use of Stimulants among Jehovah’s Witnesses

**DOI:** 10.3390/ijerph18105049

**Published:** 2021-05-11

**Authors:** Klaudia Jakubowska, Paweł Chruściel, Krzysztof Jurek, Michał Machul, Aneta Kościołek, Beata Dobrowolska

**Affiliations:** 1 Department of Development in Nursing, Faculty of Health Sciences, Medical University of Lublin, 20-081 Lublin, Poland; klaudia.jakubowska@umlub.pl (K.J.); pawel.chrusciel@umlub.pl (P.C.); aneta.kosciolek@umlub.pl (A.K.); 2 Institute of Sociology, Faculty of Social Sciences, John Paul II Catholic University, 20-950 Lublin, Poland; kjurek@interia.eu; 3 Department of Management in Nursing, Faculty of Health Sciences, Medical University of Lublin, 20-081 Lublin, Poland; mm.machul@gmail.com

**Keywords:** religiosity, attitudes, health, disease, death, alcohol drinking, cigarettes smoking, Jehovah’s Witnesses

## Abstract

Religiosity is considered as one of the many factors shaping an individual’s health consciousness. The aim of the study is an analysis of the correlation between the religiosity of Jehovah’s Witnesses and their attitudes towards health and disease. A cross-sectional study was performed on the convenience sample of 171 Jehovah’s Witnesses from eastern Poland with the use of two research tools: the author’s questionnaire, focusing on attitudes towards health, disease, death and the use of stimulants, and the Duke University Religion Index (DUREL). The research involved 99 females (57.9%) and 72 males (42.1%), with an average age of 37.25 (SD = 12.59) years. On average, they have been a Jehovah’s Witness for 29.21 (SD = 13.22) years and are characterised by a high ratio of organisational religious activity (ORA) (M = 5.60; SD = 0.62) and intrinsic religiosity (IR) (M = 4.81; SD = 0.37). Those who had never smoked before becoming one of Jehovah’s Witnesses had a higher IR (Z = −2.822; *p* = 0.005), similarly to those respondents who smoked cigarettes before they became Jehovah’s Witnesses (Z = −2.977; *p* = 0.003) and those who did not abuse alcohol before they became Jehovah’s Witnesses (Z= −1.974; *p* = 0.048). Jehovah’s Witnesses are a group characterised by a high degree of consistency when it comes to religiosity, attitudes regarding health and disease and health behaviours. This means that they follow the teachings of their religion with regard to health issues. Knowledge about the association between religiosity and health behaviours is important to provide effective health education, health promotion and development of health prevention policy, specifically when dealing with more religious groups of clients.

## 1. Introduction

Religious beliefs encompass an important aspect of human activity in the context of respect for one’s life and health [1,2]. To date, the exploration of the concept of religiosity in the context of health has covered many religious groups, and such analyses have been undertaken by representatives of various disciplines and medical specialities [3,4,5,6,7,8,9,10].

Religiosity impacts the personal concept of health and illness, and this, in turn, affects peoples’ lifestyle and health behaviours. According to research findings, this influence is usually positive, with religiosity exerting a favourable impact by encouraging healthier lifestyles and avoidance of undesirable behaviours among adolescents, young people and adults [11,12,13]. Therefore, religiosity may be regarded as a major factor when it comes to public health through its ability to discourage unhealthy behaviours [10]. Among many examples, religiosity has been documented as a protective factor associated with delayed onset of alcohol use and reduced frequency of drinking [14]; religiosity is associated with both pro-health behaviours (avoiding smoking, alcohol abuse, and regular sport activity) and emotional well-being (reflected by happiness, life satisfaction, and the sense of meaning and purpose) [10]. Moreover, religious practices and spiritual beliefs have been documented as influencing coping mechanisms in dealing with chronic illnesses, e.g., in the study by Abu et al. [15], higher levels of religiosity imply a higher level of quality of life among patients with cardiovascular diseases. Additionally, religiosity has been found to act as a moderating factor between a chronic medical condition and psychological well-being among Muslims [16]. In the study by Roger and Hatala [17], religiosity is correlated with a variety of health outcomes and serves as a personal source of hope and a coping strategy. Studies in China have shown that among Muslims with more pronounced religiosity, a lower tendency to depression was registered [18]. Similar findings were found among Mormon communities [13,19]. According to Zimmer et al. [20], there are three main mechanisms for the positive effects of religion on health. First, through social support received from the group of people linked by common values. Second, through messages underlying religious principles regarding healthy lifestyle and negative views about the use of stimulants or risky sexual behaviour. Third, some religious practices such as meditation or prayer help to reduce stress and strengthen coping mechanisms. Therefore, analysing the religiosity of different religious groups and how it is related to health behaviours is important from the perspective of prophylaxis and preventive measures [10].

Jehovah’s Witnesses are a Christian religious organisation dating back to the late 1800s. Since then, the number of Witnesses around the world has increased significantly. At the moment, they are present in 240 countries, and in 2018, the highest number of preachers (individuals identifying themselves as Jehovah’s Witnesses) was estimated at 8,579,909 in 119,954 congregations. Jehovah’s Witnesses regard the Bible as the standard for all their beliefs and practices. The Bible serves as the basis for the Watchtower Society’s literature from which members of the congregation can shape their religious consciousness and derive guidelines concerning their lifestyle, family relations, life in society, politics and medicine, as well as views related to health or disease [21,22,23].

In the organisation of Jehovah’s Witnesses, religiosity plays an important role and influences particular views, including the understanding of disease and its related aspects. The attitudes of Jehovah’s Witnesses have aroused numerous controversies and debates on how medical and nursing personnel can possibly influence their decisions about voluntary treatment. Modern medicine considers many of the views upheld by Jehovah’s Witnesses to be illogical and inconsistent. This applies, for example, to the refusal of blood transfusion accompanied by the simultaneous recognition that one’s decision, made in the name of religion, may indeed be harmful to health and even life [24,25]. 

To our best knowledge, none of the published scientific studies involving Jehovah’s Witnesses included any measurements of religiosity in the context of health, disease, death and the use of stimulants. It is worth noting that the available literature mainly employed analyses of medical records or case descriptions, and there is scarce research through direct contact with Jehovah’s Witnesses using a survey questionnaire [26,27,28]. Additionally, the use of stimulants, mostly alcohol consumption and cigarette smoking among adolescents and young adults in Poland, is reported as high, which is a basis for the recommendation of different prophylactics programs [29]. Therefore, investigating the determinants of health behaviours of different groups of inhabitants, including the influence of religiosity, may help to plan and tailor such programs according to their needs. 

## 2. Materials and Methods

### 2.1. Aim

The aim of this study is to analyse the correlation between the religiosity of Jehovah’s Witnesses and their attitudes towards health, disease, death and the use of stimulants.

### 2.2. Design

A cross-sectional study conducted in 2017 and 2018 in accordance with the Strengthening the Reporting of Observational studies in Epidemiology guidelines (STROBE) [30].

### 2.3. Study Participants

A convenience sampling method was used. The studied group was made up of members of Jehovah’s Witnesses congregations from eastern Poland. According to the Central Statistical Office, in 2018, there were 91 congregations of Jehovah’s Witnesses in this region of Poland (Lubelskie Voivodship) with 8345 followers, representing 7.13% of the population of Jehovah’s Witnesses in Poland [31]. The following inclusion criteria were applied: informed consent to participate in the study, minimum age of 18 (legal capacity), having been baptised as a Jehovah’s Witness and Polish nationality.

### 2.4. Instruments

The following instruments were used:

(a) The author’s research tool measuring attitudes towards health and disease. The instrument was developed during two stages: an analysis of the literature on the beliefs of Jehovah’s Witnesses and research into health behaviours and the impact of religion on health [2,10,27,28,32,33,34]; and consultation with two Jehovah’s Witnesses regarding their views on health, disease and medical care issues. The questionnaire consists of 65 questions in total and is divided into the following sections: sociodemographic characteristics; the attitudes of Jehovah’s Witnesses to health, disease and death; the use of stimulants; the attitudes of Jehovah’s Witnesses on selected medical procedures; the expectations of Jehovah’s Witnesses towards medical personnel. Most of the questions were based on a 5-point Likert scale (from total acceptance to total disapproval); some of the items were multiple-choice questions, and some dichotomous (yes/no) or with three possible answers (yes/no/no opinion). The questionnaire was piloted on 10 Jehovah’s Witnesses, and their opinion was taken into account in order to correct any ambiguous questions. 

This article is based on the research material collected from answers to 34 questions of the questionnaire: on the attitudes of Jehovah’s Witnesses towards health, disease, death and the use of stimulants. 

(b) The Polish version of the Duke University Religion Index (PolDUREL) as adapted in 2016 for research conducted in Poland [35]. It consists of 5 questions to assess the three main aspects of religiosity: ORA—organisational religious activities (public religious activities such as celebrating worship or participating in other religious activities related to the group); NORA—non-organisational religious activities (private religious activities such as prayer, scripture study or listening to religious radio/television stations); IR—intrinsic religiosity (personal religious engagement or motivation). ORA and NORA are rated on a 6-point Likert scale, while IR is rated on a 5-point Likert scale. The total score is between 5 and 27 points. The PolDUREL has high internal consistency (Cronbach’s alpha 0.86–0.91) and reliability (Cronbach’s alpha 0.90–0.99) [35].

### 2.5. Data Collection

The material was collected using the snowball sampling method [36]. This specific method was selected due to difficulties in reaching out to respondents. All “mass” activities related to the Jehovah’s Witnesses organisation are supervised and consulted with the elders of the congregation or a selected branch office. To collect data, the authors contacted several people who belonged to one of the congregations of Jehovah’s Witnesses.

A pencil-and-paper self-administered questionnaire was used. The data were collected by two prepared researchers, who gave each respondent an envelope with the questionnaire, and after completion, they were asked to return the questionnaire in a closed, anonymous envelope to the researchers. The questionnaire took approximately 20 minutes to complete. The aim of the study and the process of data collection was explained to those who agreed to participate. After completing the questionnaire, the respondents were asked to suggest any other believers who could be contacted and invited to participate in the study.

Considering the number of Jehovah’s Witnesses in Lublin Voivodship (8345 in 2018 [31]), the minimum number of respondents was estimated at 367 (with a maximum error of 5% and a confidence level of 95%). A total of 380 questionnaires were distributed among believers, and out of 280 returned questionnaires (73.68% response rate), 171 were qualified for the analysis as correctly filled in with all the questionnaire items in order.

### 2.6. Ethical Issues

The Bioethics Committee at the Medical University of Lublin approved the study in accordance with the requirements of the Helsinki Declaration (Decision no. KE-0254/236/2017). Each of the survey participants was informed of the purpose of the study, its voluntary nature and anonymity. All the respondents who met the criteria for inclusion in the study provided their consent, which they confirmed in writing. Consent to take part in the study was collected in a separate envelope to avoid identification of those who returned a completed questionnaire. Respondents were also informed of the possibility of withdrawal at any time of the data collection process.

### 2.7. Data Analysis

Statistical analysis was carried out with the IBM SPSS Statistics package (v.25, IBM, Krakow, Poland). For variables, the number of categories and their percentage is given. Quantitative variables are described using descriptive statistics: mean and standard deviation. Two groups were compared with the Mann–Whitney U test. Correlations between analysed variables were checked using Spearman’s rho coefficient. The obtained results of the analysis were assumed to be statistically significant at *p* < 0.05. For the material collected with the DUREL scale, the authors of the original version of the instrument recommend analysing three subscale results independently in separate regression models when their correlation with health is to be examined. The higher mean scores indicated greater ORA, NORA, and IR results [37].

The linear regression (the enter method) was used to identify variables that predict the attitudes toward health and disease. The demographic variables and religion-related variables were independent variables in the analysis. All independent variables were entered into the equation at the same time. For the purpose of this analysis, dummy coding was used for qualitative variables such as gender. Multicollinearity was checked by VIF (variance inflation factor). The Durbin–Watson test was used to detect autocorrelation in the residuals. For the goodness of model fit, R-squared and overall F-test were considered.

## 3. Results

### 3.1. Participants

The study group consisted of 171 people, where 99 (57.9%) were female, and 72 (42.1%) were male. The age of the respondents ranged from 18 to 72 years (M = 37.25; SD = 12.59). The majority of the respondents were in marital relationships (127; 74.3%) and lived in urban areas (142; 83.0%). Respondents’ membership in the Jehovah’s Witnesses organisation lasted on average 29.21 years (SD = 13.22). The studied group was also characterised by a high ratio of organisational religious activity (M = 5.60; SD = 0.62) and intrinsic religiosity (4.81; SD = 037) (Table 1).

### 3.2. Religiosity vs. Attitudes towards Health and Disease

The higher the ORA (rho = −0.170; *p* = 0.026) and IR (rho = −0,167; *p* = 0.029) ratios, the less the respondents were likely to agree with the statement that they “follow physicians’ recommendations resulting from their health situation or regular appointments for medical examinations”. In turn, higher NORA scores were accompanied by more willingness to obtain medical information and understand the cause of one’s health condition (rho = 0.206; *p* = 0.007) and less willingness to bear full responsibility for one’s health (rho = −0.180; *p* = 0.019). The ORA results were positively correlated with the belief that the disease is a misfortune that can fall on anyone (rho = 0.151; *p* = 0.049), and negatively correlated with the statement, “in the face of disease of a relative I would be willing to turn to a support group” (rho = −0.157; *p* = 0.040) (Table 2).

### 3.3. Predictors of Attitude towards Health and Disease

On the basis of questions related to health and disease, three main attitudes towards therapies were created: (a) positive attitude towards therapies recommended by physicians, (b) internal locus of control of health and (c) the belief in the impact of force majeure on health. Statistical analysis showed that only in the case of “positive attitude towards therapies recommended by physicians" the model fitted the data well (F = 4.592; *p* < 0.001). The Durbin–Watson statistic was slightly lower than 2. The model explained about 17% of the variability of the dependent variable. “A positive attitude towards therapies recommended by physicians” was positively associated with NORA (beta = 0.364; *p* < 0.001) and negatively with ORA (beta = − 0.238; *p* < 0.05). This means that people with a higher level of ORA were less positive about the treatment recommended by physicians. On the other hand, people with a high level of NORA were characterised by a more positive attitude to the treatment recommended by physicians. Moreover, females demonstrated a more positive attitude towards therapies proposed by physicians (beta = 0.161; *p* < 0.05) (Table 3).

### 3.4. Religiosity and the Importance of Prayer in the Face of Disease

NORA and IR ratios correlate positively with the statement “I usually pray at least once a day” (NORA: rho = 0.276; *p* < 0.001; IR: rho = 0.217; *p* = 0.004). IR index was positively correlated with the statement “I know the importance of prayer in difficult life situations, and I can support this with biblical examples of preventive behaviour” (rho = 0.172; *p* = 0.024) (Table 4).

### 3.5. Religiosity and Attitudes towards Death

The stronger ORA, the less interest in a support group in the face of the death of a relative (rho = −0198; *p* = 0.009) and the stronger the feeling that death is a misfortune that can fall on anyone (rho = 0.222; *p* = 0.004). Along with an increase in the level of NORA, the respondents were more likely to feel that the entire person dies at the moment of death (rho = 0.166; *p* = 0.030). The stronger IR, the more willing the respondents are to benefit from the support of their fellow congregation members (rho = 0.184; *p* = 0.016) and the feeling that death is a misfortune that can fall on anyone (rho = 0.159; *p* = 0.038) (Table 5).

### 3.6. Religiosity and the Use of Stimulants

Those who had never smoked before becoming one of Jehovah’s Witnesses had a higher IR (Z = −2.822; *p* = 0.005), similarly to those respondents who smoked cigarettes before they became Jehovah’s Witnesses (Z = −2.977; *p* = 0.003) and those who did not abuse alcohol before they became Jehovah’s Witnesses (Z = −1.974; *p* = 0.048) (Table 6).

## 4. Discussion

Considering three dimensions of religiosity, Jehovah’s Witnesses obtained high scores in IR (4.81, SD = 0.37) and ORA (M = 5.60; SD = 0.62) results. The ORA result in the surveyed group is consistent with the structure and functioning of Jehovah’s Witnesses. The members are required to meet twice a week. Larger meetings are held twice a year [38]. Furthermore, the members of the organisation are known around the world for telling other people about their beliefs [39]. Relating this result to other research results on religiosity measured by DUREL, in different religious groups, ORA scored high also among Catholic medical students (4.07; SD = 1.24) [35]. This is in contrast to Muslim students and Muslim residents, where ORA score was on a lower level (2.92, SD = 1.07 and 3.17, SD = 1.75, respectively); the same was true for Buddhist students and Buddhist residents (2.03, SD = 0.84 and 1.87, SD = 1.06, respectively) [40].

Referring to the respondents’ attitudes towards health and disease, it has been shown that higher levels of ORA and IR are associated with a weaker adherence to medical recommendations resulting from one’s health situation and regular reporting for medical examinations. These results may be explained by the fact that Jehovah’s Witnesses subordinate their entire lives primarily to God. In the face of a life-threatening situation or when proposed with a therapeutic solution (e.g., involving blood), God’s command will be more important than recommendations of a doctor or medical staff [41]. However, it does not mean that Jehovah’s Witnesses are not interested in keeping their health and life in good condition. On the contrary, they are obliged to take care of their body and avoid unhealthy behaviours such as smoking or drinking alcohol, which are unacceptable [42,43]. Similar pro-health related behaviours based on church teachings are confirmed in the studies among the Mormon community [13,19].

The higher the NORA ratio, the more often Jehovah’s Witnesses tend to seek medical information and understand the causes of their health condition, and also the less often they believe that they themselves bear full responsibility for their health. The NORA index consists of religious activities performed in private, such as praying, studying the Bible and watching or listening to religious television and radio stations [37]. The duties of each Jehovah’s Witness include the regular personal study of the Bible. Devoting time to personal religious activities constitutes a very important part of every Jehovah’s Witness’s life [44].

The higher the ORA, the less willing to seek help from a support group when a relative is ill or dying. However, respondents are willing to turn to their congregation members for support, which positively correlates with IR. Jehovah’s Witnesses are encouraged in their literature to develop a positive attitude in difficult life situations and to seek advice from their fellow brothers and sisters before making any major decisions [45]. Moreover, the structure of the entire religious community allows its members to cope with difficulties within the organisation. When faced with problems, Jehovah’s Witnesses immediately organise assistance, from financial resources, practical assistance, and medical care, to spiritual and emotional support [46]. The aspect of support received in a religious group, and the use of religiosity in coping with chronic illness and adherence to treatment is underlined in the literature [20] and confirmed in other studies [13,47,48].

Obviously, the higher the NORA and IR results, the more important prayer is in the event of disease. Jehovah’s Witnesses respect the biblical model of prayer, which is of fundamental importance to them [49]. The importance of prayer in chronic disease was confirmed by other authors [47].

The analysis of respondents’ attitudes towards stimulants shows that the majority of them have a negative attitude towards the use of stimulants, with 90.6% of respondents having never smoked since they became Jehovah’s Witnesses and 73.7% having never abused alcohol since that time. The principles regarding nutrition and the use of stimulants are derived from religious motives and have their basis in the Bible. For Jehovah’s Witnesses, smoking and alcohol abuse are habits that do not please God [50,51]. This confirms once again how Jehovah’s Witnesses identify themselves with biblical content and how much they are in agreement, as a religious community, in this regard [22,52]. A similar perspective is visible in the studies among Mormons, for whom the “body is a temple” and “a gift of God”, and therefore they feel a strong responsibility to take care of the body and practice a healthy lifestyle [13].

There are many studies that confirm the correlation of religiosity with alcohol consumption and smoking. Lin et al.’s [14] study results indicated that religiosity plays an important role in preventing/delaying the initiation and re-initiation of alcohol use and persistence of alcohol consumption. Furthermore, Francis et al. [11], in their study among learners, report that religiosity was associated with lower odds of reported alcohol and other drug use and risky sexual behaviours. A study conducted in the Polish population confirms that religiosity measured by the level of religious service attendance may have a preventive effect on smoking and alcohol abuse, particularly for the most religious people [10]. This is in line with the study by Svensson et al. [12], in which church/mosque attendees had significantly healthier dietary patterns and were less likely to be daily smokers. Additionally, people who are more involved in communal religious life (including Jehovah’s Witnesses) are less likely to be heavy drinkers [53]. In the current study, a higher IR was reported in those who had never smoked and abused alcohol before becoming Jehovah’s Witnesses and in those who smoked before becoming Jehovah’s Witnesses. IR was reported to be linked with less alcohol consumption in a study by Pelletier-Baldelli et al. [54] and with smoking status in a study by Martinez et al. [55].

### Limitations of the Study

The study has some limitations. First, the convenience sampling method, as used in the research, results in an indeterminate probability of representativeness. In addition, the exclusive nature of the Jehovah’s Witnesses community restricted the possibility of studying the same population in larger numbers. An important limitation stems from the instrument adopted in our research, the author’s own questionnaire and the fact that it was used for the first time, without validation. Moreover, collected material included mostly subjective data and was analysed using simple descriptive analysis. Another important aspect is the lack of a control group of people with different beliefs and religious affiliations. The availability of results from two or more different groups may allow comparative analysis, and so this is recommended for future research. Additionally, future studies should also address the possible negative influence of religion on Jehovah’s Witnesses health, especially in aspects related to mental well-being.

## 5. Conclusions

Religiosity has been proven to be an important factor that shapes attitudes towards various health-related aspects of human life as well as health behaviours. This is also true with respect to Jehovah’s Witnesses. Our study revealed that three main dimensions of religiosity are related to Jehovah’s Witnesses’ attitudes towards selected aspects of health, disease, death and also towards cigarette smoking and alcohol abuse. Moreover, it was found that Jehovah’s Witnesses are a group characterised by a high degree of consistency between their religiosity, attitudes towards health and disease and health behaviours. This means that with regard to health issues, they follow the teachings of their religion. In order to better understand the protective effect of religion and religiosity in regards to health behaviours of Jehovah’s Witnesses, especially towards alcohol abuse and cigarette smoking, further research of a more qualitative nature is recommended. The strong association between religiosity and health behaviours is important (as is also underlined in the literature) for the successful delivery of health education, health promotion and development of health prevention policy, specifically when dealing with more religious groups of clients.

## Figures and Tables

**Table 1:** Participants (*n* = 176).

Variables		*n*	%
Gender	female	99	57.9
	male	72	42.1
Marital status	single	34	19.9
	married	127	74.3
	divorced	5	2.9
	widowed	5	2.9
Place of residence	urban area	142	83.0
	rural area	29	17.0
		M	SD
Age		37.25	12.59
Membership in the Jehovah’s Witnesses organisation		29.21	13.22
Religiosity (DUREL scores)	ORA	5.60	0.62
	NORA	5.17	0.85
	IR	4.81	0.37

Legend: M—mean, SD—standard deviation; ORA—organisational religious activity, NORA—non-organisational religious activity, IR—internal religiosity.

**Table 2:** Attitudes towards health and disease and the dimension of religiosity.

Attitude Towards Health and Disease	Yes	Don’t Know	No	ORA		NORA		IR	
	*N* (%)			rho	*p*	rho	*p*	rho	*p*
I follow physician’s recommendations resulting from my health situation	168 (98.2)	2 (1.2)	1 (0.6)	−0.170 *	0.026	−0.016	0.838	−0.167 *	0.029
I regularly report for medical examinations	155 (90.6)	2 (1.2)	14 (8.2)	−0.204 **	0.007	0.101	0.190	−0.017	0.825
I try to obtain medical information and understand the causes of my health condition	168 (98.2)	1 (0.6)	2 (1.2)	−0.022	0.774	0.206 **	0.007	−0.073	0.342
If I get sick, I recover on my own, I am self-reliant	80 (46.8)	22 (12.9)	69 (40.3)	−0.052	0.497	−0.023	0.766	−0.034	0.663
My health is affected by random events, it is a matter of fate	128 (74.9)	10 (5.8)	33 (19.3)	−0.080	0.298	0.032	0.673	−0.142	0.064
I bear full responsibility for my health	157 (91.8)	4 (2.3)	10 (5.8)	−0.090	0.244	−0.180 *	0.019	0.063	0.413
My health depends only on how well I take care of myself	71 (41.5)	7 (4.1)	93 (54.4)	−0.032	0.678	−0.011	0.888	0.075	0.327
God has the power to cure diseases	171 (100.0)	0 (0.0)	0 (0.0)	-	-	-	-	-	-
Disease is a misfortune that can fall on anyone	171 (100.0)	0 (0.0)	0 (0.0)	0.151 *	0.049	−0.014	0.859	0.136	0.076
I understand views about disease and death and can support them with biblical evidence	169 (98.8)	0 (0.0)	2 (1.2)	0.113	0.142	0.020	0.792	−0.004	0.954
In the face of disease of a relative I would be willing to turn to a support group	117 (68.4)	22 (12.9)	32 (18.7)	−0.157 *	0.040	−0.086	0.264	0.071	0.359
In the face of disease of a relative I would be willing to turn to fellow brothers and sisters in the congregation	162 (94.7)	7 (4.1)	2 (1.2)	−0.033	0.667	0.078	0.309	0.087	0.258

Legend: *p*—significance level, rho—Spearman’s correlation coefficient, ORA—organisational religious activity, NORA—non-organised religious activity, IR—intrinsic religiosity; * *p* < 0.05; ** *p* < 0.01.

**Table 3:** Predictors of attitude towards health and disease.

Model	Attitudes towards Health and Disease					
	Positive Attitude towards Therapies Recommended by Physicians		Internal Locus of Control of Health		The Belief in the Impact of Force Majeure on Health	
	B	Beta	B	Beta	B	Beta
ORA	−0.184	−0.238 *	−0.120	−0.100	0.068	0.081
NORA	0.205	0.364 **	−0.058	−0.066	0.006	0.010
IR	−0.163	−0.125	0.110	0.054	−0.097	−0.070
Age	0.003	0.088	−0.006	−0.097	0.001	0.018
Gender ^a^	0.155	0.161 *	0.047	0.032	0.085	0.082
Marital status ^b^	−0.049	−0.045	−0.063	−0.037	0.044	0.038
Place of residence ^c^	−0.029	−0.023	−0.120	−0.100	−0.237	−0.174
F	4.592; *p* < 0.001		0.715; *p* = 0.659		1.185; *p* = 0.314	
R	0.41		-		-	
R^2^	0.17		-		-	

B—unstandardised coefficient; Beta—standardised coefficient; F—statistic for linear regression; R—the coefficient of correlation; R^2^—the coefficient of determination; ^a^ 1—female; 0—male; ^b^ 1—single; 0—married; ^c^ 1—urban area; 0—rural area; * *p* < 0.05; ** *p* < 0.001.

**Table 4:** The importance of prayer in disease.

The Importance of Prayer in Disease	Yes	Don’t Know	No	ORA		NORA		IR	
	*N* (%)			rho	*p*	rho	*p*	rho	*p*
I usually pray at least once a day	162 (94.7)	3 (1.8)	6 (3.5)	−0.073	0.342	0.276 **	<0.001	0.217 **	0.004
In the face of physical disease, I pray to God	165 (96.5)	4 (2.3)	2 (1.2)	−0.035	0.648	0.124	0.106	0.123	0.110
In the face of disease, I would pray to God for the spiritual strength needed to remain loyal in such a situation	167 (97.7)	3 (1.8)	1 (0.6)	0.000	0.999	0.063	0.415	0.125	0.103
I know the importance of prayer in difficult life situations and I can support this with biblical examples	168 (98.2)	3 (1.8)	0 (0.0)	−0.075	0.333	0.054	0.482	0.172 *	0.024

Legend: *p*—significance level, rho—Spearman’s correlation coefficient, ORA—organisational religious activity, NORA—non-organised religious activity, IR—intrinsic religiosity; * *p* < 0.05; ** *p* < 0.01.

**Table 5:** Religious views and respondents’ attitudes towards death.

Attitude towards Death	Yes	Don’t Know	No	ORA		NORA		IR	
	*N* (%)			rho	*p*	rho	*p*	rho	*p*
At the moment of death, the entire person dies (the human soul and body are interchangeable terms)	171 (100.0)	0 (0.0)	0 (0.0)	−0.075	0.333	0.166 *	0.030	0.019	0.804
In the face of death of a relative I would be willing to turn to a support group	111 (64.9)	29 (17.0)	31 (18.1)	−0.198 **	0.009	−0.089	0.246	0.078	0.312
In the face of death of a relative I would be willing to turn to fellow brothers and sisters in the congregation	165 (96.5)	5 (2.9)	1 (0.6)	−0.055	0.472	0.054	0.485	0.184 *	0.016
Death is a misfortune that can fall on anyone	170 (99.4)	0 (0.0)	1 (0.6)	0.222 **	0.004	−0.004	0.957	0.159 *	0.038

Legend: *p*—significance level, rho—Spearman’s correlation coefficient, ORA—organisational religious activity, NORA—non-organised religious activity, IR—intrinsic religiosity; * *p* < 0.05; ** *p* < 0.001.

**Table 6:** The attitude of Jehovah’s Witnesses towards the use of stimulants.

Attitude towards Death		*N* (%)	ORA		NORA		IR	
			M	SD	M	SD	M	SD
I don’t smoke because I know the health consequences of this	No	5 (2.9)	5.80	0.45	5.20	0.84	5.00	0.00
	Yes	166 (97.1)	5.69	0.62	5.17	0.85	4.81	0.37
Statistics			Z = −0.271 *p* = 0.787		Z = −0.010 *p* = 0.992		Z = −1.353 *p* = 0.176	
I have never smoked a cigarette since I became a Jehovah’s Witness	No	16 (9.4)	5.75	0.45	4.94	0.93	4.69	0.37
	Yes	155 (90.6)	5.68	0.63	5.19	0.84	4.82	0.36
Statistics			Z = −0.043 *p* = 0.966		Z = −1.144 *p* = 0.253		Z = −2.822 *p* = 0.005	
I smoked cigarettes before I became a Jehovah’s Witness	No	118 (69)	5.58	0.69	5.09	0.84	4.69	0.44
	Yes	53 (31)	5.74	0.58	5.20	0.85	4.86	0.31
Statistics			Z = −1.637 *p* = 0.102		Z = −0.982 *p* = 0.326		Z = −2.977 *p* = 0.003	
I have never abused alcohol since I became a Jehovah’s Witness	No	45 (26.3)	5.67	0.71	5.09	1.02	4.79	0.40
	Yes	126 (73.7)	5.70	0.58	5.20	0.78	4.82	0.36
Statistics			Z = −0.163 *p* = 0.871		Z = −0.264 *p* = 0.792		Z = −0.173 *p* = 0.862	
I abused alcohol before I became a Jehovah’s Witness	No	120 (70.2)	5.73	0.62	5.19	0.82	4.86	0.32
	Yes	51 (29.8)	5.59	0.61	5.12	0.91	4.71	0.44
Statistics			Z= −2.095 *p* = 0.036		Z = −0.392 *p* = 0.695		Z = −1.974 *p* = 0.048	

Legend: *p*—significance level, Z—Mann–Whitney U test; M—mean value, SD—standard deviation.

## Data Availability

The data presented in this study are available on request from the corresponding author. The data are not publicly available due to the protection of the privacy of research subjects.
